# Alterations of global histone H4K20 methylation during prostate carcinogenesis

**DOI:** 10.1186/1471-2490-12-5

**Published:** 2012-03-13

**Authors:** Turang E Behbahani, Philip Kahl, Johannes von der Gathen, Lukas C Heukamp, Claudia Baumann, Ines Gütgemann, Bernhard Walter, Ferdinand Hofstädter, Patrick J Bastian, Alexander von Ruecker, Stefan C Müller, Sebastian Rogenhofer, Jörg Ellinger

**Affiliations:** 1 Klinik und Poliklinik für Urologie und Kinderurologie, Universitätsklinikum Bonn, Bonn, Germany; 2 Institut für Pathologie der Universität zu Köln, Cologne, Germany; 3 Institut für Pathologie, Universitätsklinikum Bonn, Bonn, Germany; 4 Urologische Universitätsklinik, Universitätsklinikum Erlangen, Erlangen, Germany; 5 Institut für Pathologie, Universitätsklinik Regensburg, Regensburg, Germany; 6 Urologische Klinik und Poliklinik, Universitätsklinikum Großhadern, Munich, Germany

**Keywords:** Histone, Methylation, H4K20, Prostate cancer, Epigenetics

## Abstract

**Background:**

Global histone modifications have been implicated in the progression of various tumour entities. Our study was designed to assess global methylation levels of histone 4 lysine 20 (H4K20me1-3) at different stages of prostate cancer (PCA) carcinogenesis.

**Methods:**

Global H4K20 methylation levels were evaluated using a tissue microarray in patients with clinically localized PCA (n = 113), non-malignant prostate disease (n = 27), metastatic hormone-naive PCA (mPCA, n = 30) and castration-resistant PCA (CRPC, n = 34). Immunohistochemistry was performed to assess global levels of H4K20 methylation levels.

**Results:**

Similar proportions of the normal, PCA, and mPCA prostate tissues showed strong H4K20me3 staining. CRPC tissue analysis showed the weakest immunostaining levels of H4K20me1 and H4K20me2, compared to other prostate tissues. H4K20me2 methylation levels indicated significant differences in examined tissues except for normal prostate versus PCA tissue. H4K20me1 differentiates CRPC from other prostate tissues. H4K20me1 was significantly correlated with lymph node metastases, and H4K20me2 showed a significant correlation with the Gleason score. However, H4K20 methylation levels failed to predict PSA recurrence after radical prostatectomy.

**Conclusions:**

H4K20 methylation levels constitute valuable markers for the dynamic process of prostate cancer carcinogenesis.

## Background

Valuable insight into epigenetics of prostate cancer (PCA) carcinogenesis offers starting points for potential new markers of non-invasive cancer diagnosis, progression and prognosis [1]. Recent observations suggest that changes in DNA methylation or gene silencing by CpG island promoter hypermethylation are not isolated events in inactivation of tumor suppressor genes, but accompanied by alterations of global histone modification levels as common hallmarks of PCA cells [2]. Histones are subject to a variety of posttranslational modifications (e.g. methylation, acetylation) on the N-terminal tail. These modifications function either by disrupting chromatin contacts or by affecting the recruitment of various proteins to the chromatin and thereby regulating transcription [3]. Histone lysine methylation results in transcriptional activation or repression, depending on the modified lysine site, and can be intensified regarding an increasing number of methylation marks (mono-, di- and tri- methylation). Histone H4 lysine 20 (H4K20) represents one of the nucleosomal core histone modifications which is involved in various cellular processes, depending on its methylation: Mono-methylated H4K20 (H4K20me1) plays a crucial role in transcriptional repression and X inactivation [4], whereas di-methylated H4K20 (H4K20me2) is linked to cellular mechanisms of DNA reparation based on its binding ability for 53BP1 at sites of double stranded DNA damages [5]. Fraga et al. demonstrated an association between reduced tri-methylated H4K20 (H4K20me3) and cancer, while it is indistinctively clarified whether the reduction of H4K20 is cause or consequence for carcinogenesis [6].

Alterations in histone H4 modifications have been shown for other cancers [7,8], especially the loss of lysine 16 acetylation and lysine 20 tri-methylation in lymphoma, lung, breast and colon cancer cells. The role of H4K20 methylation has not been investigated sufficiently in genitourinary cancers. While Schneider et al. have demonstrated that global histone tri-methylation levels of H4K20 predict cancer-specific survival in bladder cancer [9], yet a systemic analysis of H4K20 methylation levels in PCA and its impact on carcinogenesis remains open. Recent studies suggest that lower levels of global histone H3 lysine 4 di-methylation - and histone H3 lysine 18 acetylation levels [10] as well as alterations in the methylation levels of further H3 (H3K4, H3K9) and H4 (H4Ac) histones [11] allow the prediction of prognosis and biochemical recurrence of PCA.

Our study investigates global H4K20 mono-, di- and tri-methylation levels using immunohistochemistry in various states of prostate carcinogenesis (i.e. clinically localized PCA, metastatic PCA and castration-resistant PCA).

## Methods

### Patients and tissue microarray

A tissue microarray was applied to study the global H4K20 methylation levels in randomly chosen tissue from our tissue bank of patients with clinically localized PCA (patients with PCA undergoing radical prostatectomy, n = 113) and non-malignant prostate disease (patients undergoing radical cystectomy (n = 5) for bladder cancer, and transurethral resection of the prostate (n = 12) or retropubic adenomectomy (n = 6) for benign prostate enlargement); the tissue microarray was described earlier [12]. All control samples underwent a haematoxylin/eosin staining to exclude the presence of cancer cells and revealed benigne prostate hyperplasia (n = 17) and normal prostate tissue (n = 6). Surgery has been performed at the departments of urology at the Universitätsklinikum Bonn and the Waldkrankenhaus Bonn between 1995 and 2002. None of the included patients underwent neoadjuvant hormonal therapy or radiation prior surgery. The median follow-up period was 29 months (range 1-140 months) which was available for 77 patients of whom 20 suffered from recurrence, defined as a single PSA measurement > 0.2 ng/ml following radical prostatectomy. See Table 1 for clinical-pathological parameters. Retrospectively, all cases were re-evaluated for histopathological staging according the UICC TNM-staging system published in 2002 and grading according the Gleason Scoring system published in 2005. The tissue microarray was prepared from formalin-fixed, paraffin-embedded tissue specimens using a manual device (Beecher Instruments, Sun Prairie, WI, USA). Three tissue cores (including the lowest and highest Gleason grade) were arrayed to obtain a representative image of the tumour. Further analyses of this study included a tissue microarray with samples of advanced PCA undergoing palliative transurethral resection of the prostate at the departments of urology at the Universitätsklinikum Erlangen and Universitätsklinikum Regensburg (also described in [12]), which were derived from patients with metastatic hormone-naive PCA (mPCA, n = 30) and castration-resistant PCA (CRPC, n = 34). This tissue microarray contained a single core of PCA tissue of each patient. The local ethic committee has approved this study (ethic vote #289/08).

**Table 1:** Clinical-pathological parameters of patients with prostate cancer and non-malignant prostate disease

	PCA (n = 113)	BPH (n = 17)	normal (n = 6)
**serum PSA**			
< 4 ng/ml	9 (7.9%)	0 (0.0%)	0 (0.0%)
4-10 ng/ml	48 (42.5%)	2 (11.7%)	0 (0.0%)
> 10 ng/ml	48 (42.5%)	2 (11.7%)	0 (0.0%)
n.a.	8 (7.1%)	13 (76.5%)	6 (100%)

**digital rectal examination**			
normal	51 (45.1%)		
suspicious	50 (44.2%)		
n.a.	12 (10.6%)		

**pathological stage**			
pT2	61 (54.0%)		
pT3a	33 (29.2%)		
pT3b	16 (14.1%)		
pT4	3 (2.6%)		
seminal vesicle infiltration	18 (15.8%)		
lymph node metastasis	9 (7.9%)		

**Gleason Score**			
< 7	48 (42.5%)		
3 + 4 = 7	11 (9.8%)		
4 + 3 = 7	11 (9.8%)		
8-10	43 (38.0%)		

n.a., not available; PCA, prostate cancer; BPH, benign prostate hyperplasia

### Immunohistochemistry

The paraffin sections (5 μm) were cut from the tissue microarray block and used within one week with a subsequent deparaffinization, using xylene and applying a rehydration with graded ethanol. Slides were placed in target retrieval solution (citrate buffer, pH 6.0) and heated for 10 minutes at boiling temperature (microwave, 600 W). Following a cooling period of 15 minutes, the endogenous peroxidase activity was blocked by treatment with 3% H2O2 for 10 minutes. Afterwards, the sections were washed with tris-buffered saline and underwent a 15 minute protein block with normal swine serum with a subsequent application of the primary antibodies overnight at 4°C [antibodies were purchased from Upstate, Lake Placid, NY, USA: H4K20me1 (dilution 1:2000; catalogue number 39176; Active Motife, Carlsbad, CA, USA), H4K20me2 (dilution 1:250; catalogue number 07-747; Upstate, Lake Placid, NY, USA), H4K20me3 (dilution 1:400; catalogue number 07-463; Upstate, Lake Placid, NY, USA)]. Immunohistochemical staining was performed using the streptavidine-biotin-peroxidase complex technique (LSAB+, DAKO Cytomation, Glostrup, Denmark). The biotin-conjugated secondary antibody was incubated 30 minutes at room temperature and the avidin biotin enzyme reagent alike. The peroxidase was developed with the AEC system (DAKO). The sections were counterstained with haematoxylin and mounted. A semi-quantitative scoring system has been introduced to evaluate immunostaining results: The number of positive epithelial cells was estimated per core and scaled (0, no positive cells; 1, 1-25% positive cells; 2, 26-50% positive cells; 3, 51-75% positive cells; and 4, 76-100% positive cells). These scores were multiplied with an intensity scale (0, negative; 1, weak; 2, moderate; and 3, intensive staining). The intensity and frequency score were multiplied and categorized into low (score: 0-4), moderate (score: 5-8) and high (score: 9-12). Three cores where evaluated in each patient with clinically localized PCA; a single core was analyzed in patients with metastatic PCA. The evaluation of immunohistochemical staining has been performed without knowledge of the clinical-pathological parameters.

### Statistical analysis

Ascertained staining scores were applied to the chi-square test to determine differences of global H4K20 methylation levels in normal prostate tissue and PCA, or various stages of PCA. A Cox proportional hazard regression analysis was used to correlate the period of biochemical recurrence free-survival following radical prostatectomy with global H4K20 methylation levels or clinical-pathological parameters. Significance was concluded at *p *< 0.05. Statistical analyses were performed using PASW Statistics 18 (SPSS, Chicago, IL, USA).

## Results

**Figure 1:**
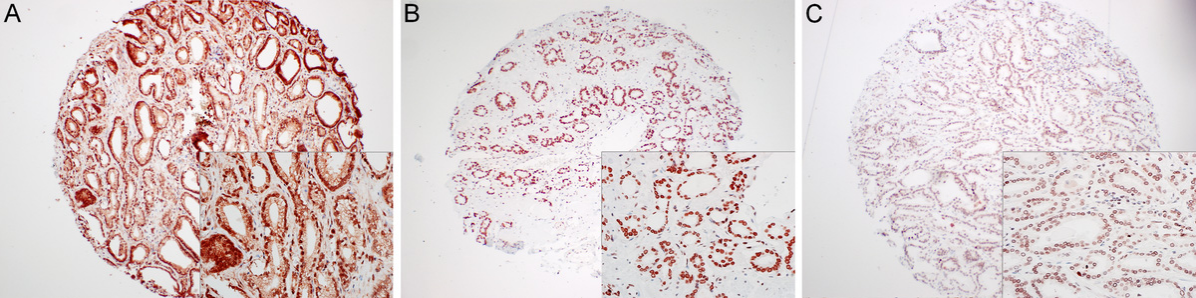
**Representative staining of H4K20me1 (A), H4K20me2 (B) and, H4K20me3 (C) in prostate cancer tissue; all antibodies are showing nuclear staining (magnification ×10; in-box figure magnified ×20)**.

As expected, we observed a nuclear staining of H4K20me1-3 (Figure 1) with different levels of H4K20 methylation at various stages of PCA. In general, immunostaining was lowest in H4K20me1, moderate to strong in H4K20me2 and moderate to high in H4K30me3.

**Figure 2:**
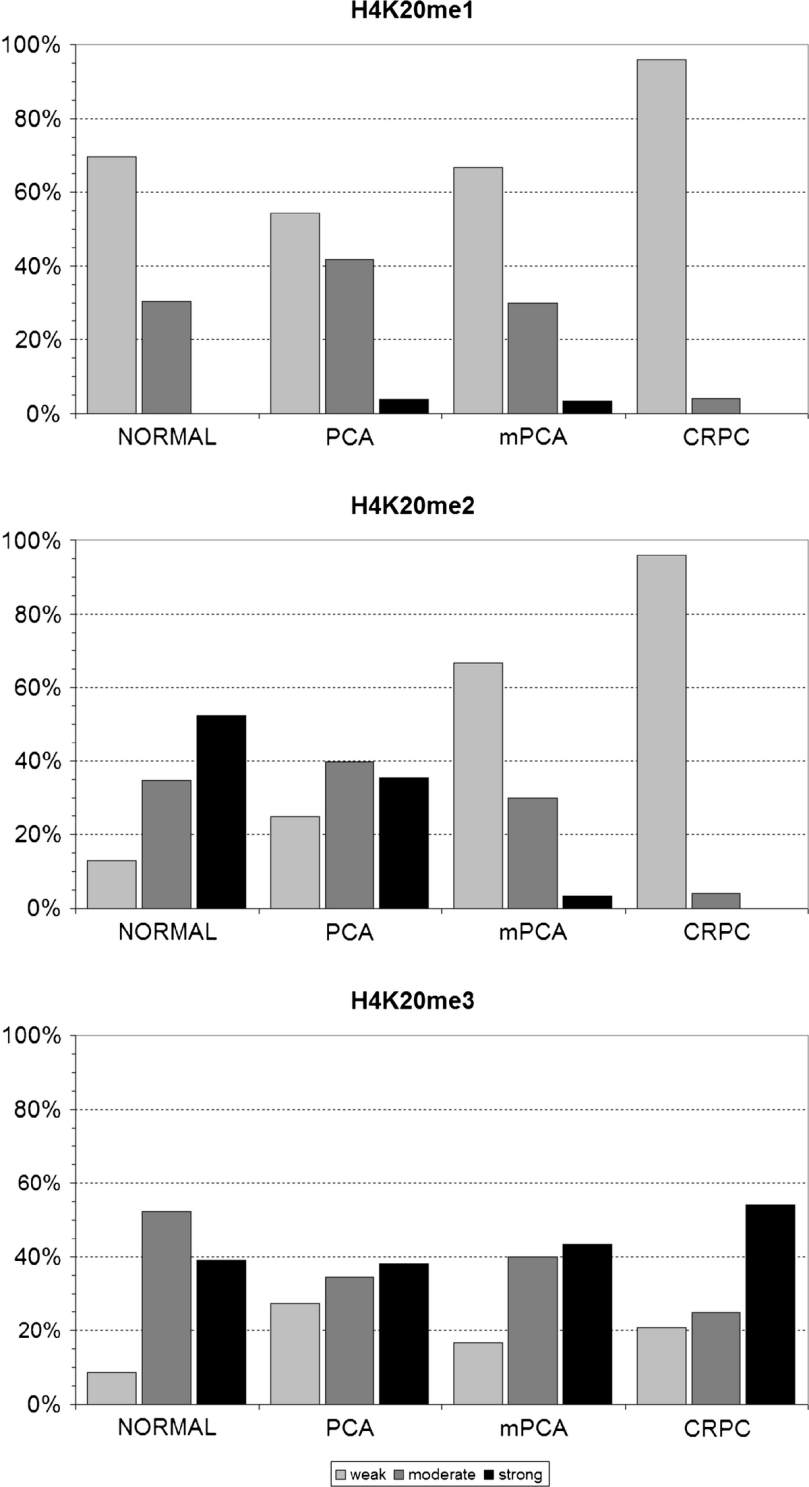
**The columns show the distribution of the different global H4K20 methylation levels in non-malignant prostate tissue (n = 23), localized prostate cancer (PCA; n = 113), metastatic hormone-naïve prostate cancer (mPCA; n = 30) and castration-resistant prostate cancer (CRPC; n = 34)**. H4K20 methylation levels were categorized into weak (≤ 4), moderate (5-8) and strong (≥ 9).

**Figure 3:**
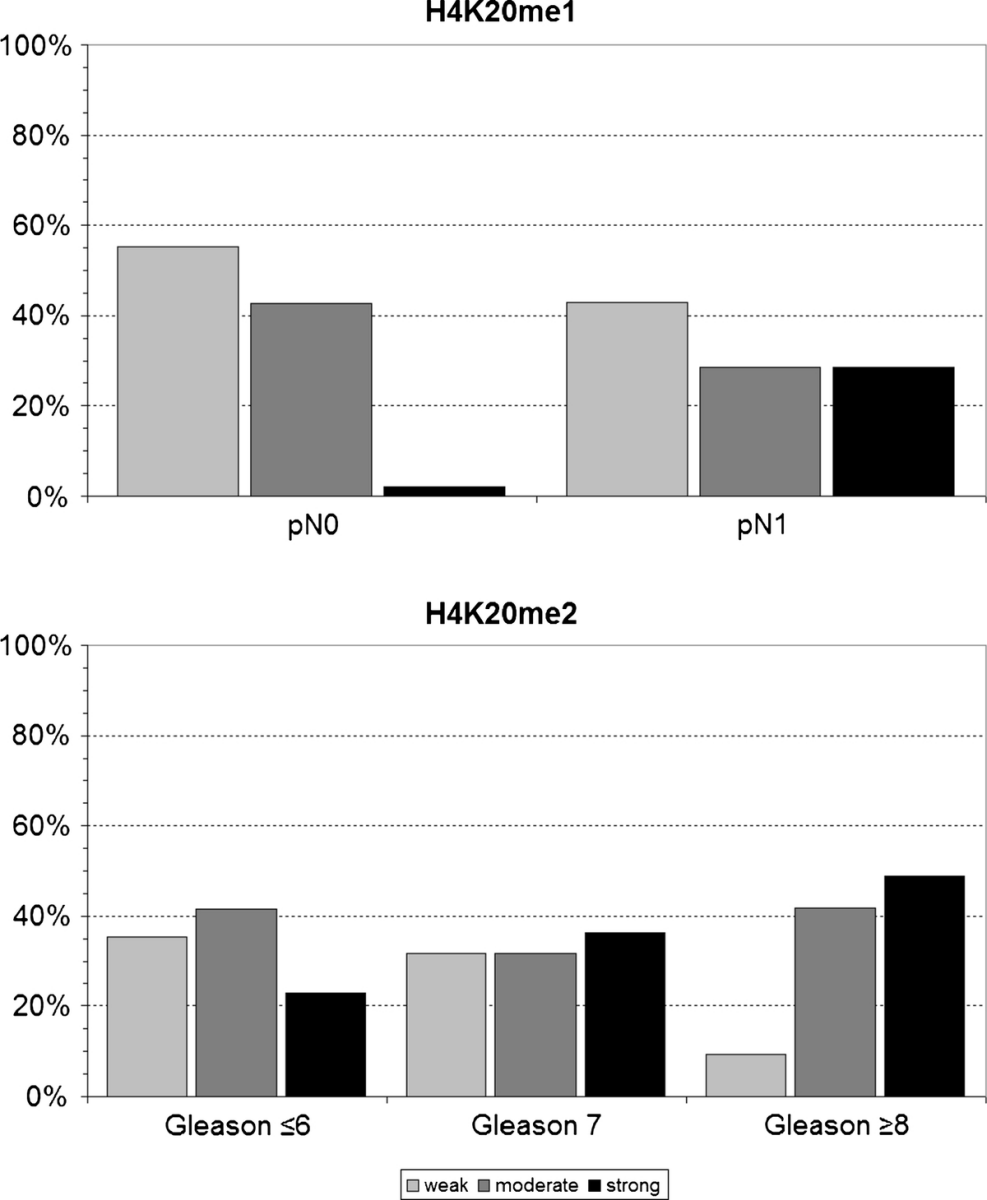
**Correlation of global H4K20 methylation levels with adverse clinical-pathological parameters: H4K20me1 was positively correlated with lymph node involvement and H4K20me2 was correlated with Gleason Score**.

*H4K20me1*. H4K20me1 staining was significantly lower in CRPC than in normal prostate tissue, localized PCA or metastatic PCA (see Figure 2 and Table 2 for details). The level of H4K20me1 was significantly increased in patients with lymph node metastases (Figure 3; Table 3). H4K20me1 was not correlated with PSA recurrence following radical prostatectomy (Table 4).

**Table 2:** Global H4K20 methylation levels are different at various stages of prostate cancer

	H4K20me1	H4K20me2	H4K20me3
NORMAL vs. PCA	*p *= 0.323	*p *= 0.261	*p *= 0.113
NORMAL vs. mPCA	*p *= 0.676	***p *< 0.001**	*p *= 0.575
NORMAL vs. CRPC	***p *= 0.017**	***p *< 0.001**	*p *= 0.136
PCA vs. mPCA	*p *= 0.483	***p *< 0.001**	*p *= 0.482
PCA vs. CRPC	***p *= 0.001**	***p *< 0.001**	*p *= 0.345
mPCA vs. CRPC	***p *= 0.030**	***p *= 0.030**	*p *= 0.509

PCA, prostate cancer; mPCA, metastatic prostate cancer; CRPC, castration resistant prostate cancer

**Table 3:** Global H4K20 methylation levels are correlated to adverse clinical-pathological parameters

	H4K20me1	H4K20me2	H4K20me3
preoperative PSA†	*p *= 0.122	*p *= 0.573	*p *= 0.151
pT-stage	*p *= 0.122	*p *= 0.573	*p *= 0.157
capsular penetration	*p *= 0.094	*p *= 0.469	*p *= 0.902
seminal vesicle infiltration	*p *= 0.127	*p *= 0.947	*p *= 0.468
lymph node metastases	***p *= 0.002**	*p *= 0.196	*p *= 0.091
Gleason Score‡	*p *= 0.191	***p *= 0.021**	*p *= 0.074

† categorized: PSA < 4 ng/ml, 4-10 ng/ml, > 10 ng/ml; ‡ categorized: Gleason Sum ≤ 6, 7, ≥ 8

**Table 4:** Cox proportional hazard regression analysis for the prediction of PSA recurrence

	significance	hazard ratio (95%CI)
preoperative PSA	*p *= 0.251	1.014 (0.991-1.037)
pT-stage	*p *= 0.764	1.127 (0.518-2.450)
capsular penetration	*p *= 0.836	0.905 (0.352-2.327)
seminal vesicle infiltration	*p *= 0.940	0.961 (0.345-2.679)
lymph node metastases	*p *= 0.448	1.541 (0.504-4.705)
Gleason Score	*p *= 0.704	0.953 (0.743-1.222)
H4K20me1	*p *= 0.858	1.068 (0.521-2.188)
H4K20me2	p = 0.385	1.311 (0.711-2.417)
H4K20me3	*p *= 0.386	1.348 (0.686-2.646)

95%CI, 95% confidence interval

*H4K20me2*. H4K20me2 was similar in normal prostate tissue and localized PCA, whereas its level was significantly reduced in tissue of patients with mPCA and CRPC. The H4K20me2 level was even lower in CRPC than in mPCA (Table 2, Figure 2). We observed a significant correlation of Gleason Score and H4K20me2 in patients with localized PCA (Table 3; Figure 2). Biochemical recurrence following prostatectomy and H4K20me2 were not correlated (Table 4).

*H4K30me3*. H4K20me3 levels were similar in normal prostate tissue, localized PCA, mPCA and CRPC (Table 2), and we did not observe an association with any clinical-pathological parameter (Table 3) no PSA recurrence (Table 4) following radical prostatectomy.

## Discussion

The importance of global histone methylation levels has first been published by Seligson et al. and has not yet been investigated for H4K20 in the view of prostate carcinogenesis and its prognosis [13]. Compared to normal prostate tissue, the present study displays an aberrant pattern of H4K20 modifications during PCA progression with a general hypomethylation of H4K20me1 and H4K20me2 in mPCA and CRPC.

While H4K20me2 is shown to sufficiently differentiate between different stages of PCA except for normal prostate tissue versus localized PCA, H4K20me1 sufficiently differentiates CRPC from other stages of PCA development. These findings suggest that modifications in histone methylation may not be applied primarily as tumour markers, rather than help to distinguish the dynamic process of cancer progress and risk assessment. Analogous findings have been reported by Schneider et al. [9] regarding the carcinogenesis of bladder cancer, with decreasing levels of H4K20me1-3 from normal urothelium over non-muscle invasive bladder cancer, muscle-invasive bladder cancer to metastatic bladder cancer. The authors concluded that H4K20me1-3 levels help identifying patients with poor prognosis after radical cystectomy

Recent studies indicate H4K20me3 to be strongly decreased during disease progression of squamous cell cancer of the lungs and, thus being an important marker for therapeutic approaches [14]. To date, it remains unanswered whether hypomethylation is cause or consequence of carcinogenesis, although recent studies suggest that dynamics in H4K20 methylation contribute to deregulations in cell cycle control and oncogenic transformation [15] which is conducive to correlations of H4K20 methylation levels with adverse clinical-pathological parameters: Interestingly, H4K20me1 was positively correlated to lymph node metastases (*p *= 0.002) which is consistent with its expression profile in mPCA. Similar results are demonstrated for H4K20me2, which shows a positive correlation to the categorized Gleason sum.

Our regression analysis did not allow the prediction of PSA recurrence after radical prostatectomy via H4K20 methylation levels. In contrast, Zhou et al. reported that H4K20me3 levels, accompanied by preoperative PSA levels, allowed risk stratification for PSA recurrence after radical prostatectomy [16]. It seems that other histone modifications are more important prognostic markers in patients with PCA: H3K4me1 [12] and H3K4me2 [10] were significant predictors of PSA recurrence following radical prostatectomy. In comparison, H3K27 methylation is correlated with many adverse clinical-pathological parameters (i.e. Gleason Score, pathological stage) but not with PSA recurrence [17]. It has to be noted that follow-up information was only available for 77 patients in our study, and also established predictive parameters like pathological stage/grade failed to correlate with PCA recurrence indicating insufficient statistical power.

Furthermore, Ellinger et al. were able to show the acteylated Histone H3 (H3Ac) and di-methylated H3 lysine 9 (H3K9me2) to be valuable discriminators between non-malignant prostate tissue and PCA with a high sensitivity (> 78%) and specificity (> 91%) [12]. Although recent studies suggest the PSA value and the PSADT to be associated with a risk development of metastatic disease in CRPC patients [18] there is no clear surrogate so far which can determine disease progression or progression after first-line chemotherapy in these patients.

Though, H4K20me3 does not allow significant differentiations between various stages of PCA it shows strong methylation levels in mPCA and CRPC, compared to H4K20me1/2 (see Figure 3). These observations might accompany recent findings by Vertino et al. [19], who describe the tri-methylation of H4K20 via SUV420H2 to play a crucial role in silencing tumor suppressor genes, such as TMS1 (target of methylation-induced silencing), which encodes for caspase recruitment domains, contributing to the promotion of apoptosis in PCA cells [20] and has been reported to be prognostic for PCA carcinogenesis [21]. Although this study does not address effects of H4K20 methylation changes on gene expression, it offers perspectives for further investigations on which mechanisms contribute to hypomethylated states in different stages of carcinogenesis. To date there are no demethylases detected [22] which are accountable for the significant reduction of H4K20me1/2 methylated states in mPCA and CRPC. The expression level of H4K20 methylases is yet no explored in PCA; however, loss of the H4K20 methylase Suv4-20h2 were associated with lower levels of H4K20me3 in a rodent-model of hepatocarcinogenesis [7] and breast cancer cell lines [8].

## Conclusions

H4K20 methylation levels constitute valuable markers for the dynamic process of prostate cancer carcinogenesis.

## Competing interests

The authors declare that they have no competing interests.

## Authors' contributions

The specimen for the tissue microarray from patients with localized PCA were selected and reviewed by PK, LCH and IG; specimen from patients with metastatic PCA were provided by BW, FH and SR. JVDG carried out the immunohistochemical experiments. JVDG and PK analyzed the tissue microarray. JVDG and JE performed the statistical analyses. The manuscript was written by TEB, CB and JE. AVR, SCM, PJB and JE participated in the study design. All authors read and approved the final manuscript.

## Pre-publication history

The pre-publication history for this paper can be accessed here:

http://www.biomedcentral.com/1471-2490/12/5/prepub
